# Effects of cold on murine brain mitochondrial function

**DOI:** 10.1371/journal.pone.0208453

**Published:** 2018-12-06

**Authors:** Matthew E. Pamenter, Gigi Y. Lau, Jeffrey G. Richards

**Affiliations:** 1 Department of Biology, University of Ottawa, Ottawa, Ontario, CAN; 2 Ottawa Brain and Mind Research Institute, Ottawa, Ontario, CAN; 3 Department of Zoology, University of British Columbia, Vancouver, British Columbia, CAN; University of PECS Medical School, HUNGARY

## Abstract

Therapeutic hypothermia is a strategy that reduces metabolic rate and brain damage during clinically-relevant hypoxic events. Mitochondrial respiration is compromised by hypoxia, with deleterious consequences for the mammalian brain; however, little is known about the effects of reduced temperature on mitochondrial metabolism. Therefore, we examined how mitochondrial function is impacted by temperature using high resolution respirometry to assess electron transport system (ETS) function in saponin-permeabilized mouse brain at 28 and 37°C. Respirometric analysis revealed that, at the colder temperature, ETS respiratory flux was ~ 40–75% lower relative to the physiological temperature in all respiratory states and for all fuel substrates tested. In whole brain tissue, the enzyme maximum respiratory rates for complexes I-V were similarly reduced by between 37–88%. Complexes II and V were particularly temperature-sensitive; a temperature-mediated decrease in complex II activity may support a switch to complex I mediated ATP-production, which is considerably more oxygen-efficient. Finally, the mitochondrial H^+^-gradient was more tightly coupled, indicating that mitochondrial respiration is more efficient at the colder temperature. Taken together, our results suggest that improvements in mitochondrial function with colder temperatures may contribute to energy conservation and enhance cellular viability in hypoxic brain.

## Introduction

Therapeutic hypothermia is a pharmacologically or physiologically (*i*.*e*. through cold exposure) induced reduction in body temperature that has proven to be an effective intervention against ischemic brain injury [1]. Beyond ischemia, therapeutic hypothermia is also protective against radiation induced damage [2], infection [3], cardiac arrest-related edema [4], neurotoxicity [5], and traumatic brain damage [6], among other pathologies. In general, therapeutic hypothermia decreases excitotoxicity during ischemia and reduces oxidative stress and subsequent initiation of cell death pathways. Interestingly, many species have evolved remarkable abilities to tolerate prolonged environmental hypoxia [7, 8], and it is notable that these species typically exhibit low basal metabolic rates and body temperatures and/or reduce their metabolic rate and body temperature set points when they experience challenging low-oxygen environments [7, 9, 10]. These species also tend to be very tolerant of clinically relevant ischemic stresses *in vivo* and *ex vivo* and exhibit minimal oxidative stress and also avoid cell death pathway initiation during ischemia [8, 11–13]. Therefore, this tolerance may be linked to thermoregulatory flexibility during low-oxygen stress.

Mitochondria are the primary source of cellular energy and their activity is thus central to the determination of metabolic rate and the generation of metabolic heat. Aerobic energy production in the brain occurs primarily via mitochondrial oxidative phosphorylation. Oxidative phosphorylation is the process by which ATP is formed as a result of substrate oxidation, which donates electrons to the electron transport system (ETS). Adaptations to this metabolic process are crucial in organisms that experience environmental hypoxia and enable such species to appropriately match cellular responses and whole animal metabolic rate to a wide range of oxygen tensions. For example, reduced ETS respiratory flux and/or mild uncoupling of the proton (H^+^) gradient is commonly observed in isolated mitochondria from various tissues of hypoxia-adapted species [14–17]. Beyond energetics, mitochondria are cellular signalling hubs that detect and integrate low oxygen signals, and then initiate either cell death cascades or neuroprotective strategies [18–21]. Therefore, mitochondria are at the center of both neuronal energy production in normoxia and also the cellular decision between initiating neuroprotective responses *vs*. activating cell death cascades when oxygen is limited.

Recently, we have demonstrated that mitochondrial function in brain is drastically down-regulated in response to acute hypoxia and chronic anoxia in hypoxia- and anoxia-tolerant species [16, 17], and that these decreases correlate with changes in whole-animal metabolic rate and/or body temperature. Given the putative interplay between mitochondrial function and metabolic rate regulation in hypoxia-tolerant species, and the efficacy of therapeutic hypothermia in presumably reducing metabolic rate and limiting cell damage in models of brain ischemia, we asked how hypoxia-intolerant brain mitochondrial ETS function is impacted by a thermal shift to therapeutic hypothermia temperatures. We are not aware of any studies examining the effects of hypoxia on mitochondrial function in human brain; however, murine models are commonly used as surrogates for human brain studies. Furthermore, brain mitochondria from humans and mice retain a similar degree of respiratory function for a similar period of time post-mortem [22], suggesting that measurements from murine brain mitochondria may be a reasonable facsimile for measurements from human brain mitochondria. We utilized BALB/c mice in our study because this strain of mouse is particularly intolerant to hypoxia [23, 24], and exhibits metabolic responses to hypoxic exposures that are more similar to those of adult humans than other common strains of mice [24, 25]. We hypothesized that the effect of temperature on mitochondrial function would scale predictably with temperature coefficient relationships (Q_10_; *i*.*e*., the temperature sensitivity of an enzymatic reaction rate due to a temperature increase of 10°C). To test this hypothesis, we compared mitochondrial respiration rates and H^+^ conductance in saponin-permeabilized murine brain tissue, and also ETS enzyme complex capacities, at a physiological temperature (37°C) and at a clinically-relevant therapeutic hypothermia temperature (28°C).

## Materials and methodology

### Ethical approval

All protocols were performed with the approval of The University of British Columbia Animal Care Committee. BALB/c Mice [*Mus musculus*, Linnaeus, 1758] were obtained from Charles River and were housed in pairs at room temperature under a 12:12 h light-dark cycle and fed rodent chow *ad libitum*. Animals were not fasted prior to experimental trials.

### Permeabilized brain mitochondrial preparation

Animals were sacrificed by cervical dislocation and whole brains were extracted over ice and bisected laterally within 30 secs. One half of each brain was frozen in liquid nitrogen and stored at -80°C for enzyme analysis (see below); the other half was placed into ice-cold homogenization buffer (in mM: 250 sucrose, 10 TrisHCl, 0.5 Na_2_EDTA; 1% fatty acid-free BSA, pH 7.4 at 4°C) and then minced over ice for 2–3 mins until the individual tissue pieces were uniformly smaller than grains of sand. The resulting homogenate was permeabilized with 4 mM saponin in homogenization buffer for 45 mins, as described previously [16]. Following permeabilization, the cell homogenate was re-suspended in ice-cold BIOPS medium (in mM: 10 Ca-K_2_-EGTA, 5.8 NaATP, 6.6 MgCl_2_, 20 imidazole, 20 taurine, 50 potassium 2-(N-morpholino)ethanesulfonic acid (K-MES), 15 Na-phosphocreatine, 0.5 dithiothreitol (DTT); pH 7.1, adjusted with 5 N KOH), rinsed for 2 mins on ice, and then re-suspended in BIOPS medium. This rinse procedure was repeated 3 times. Permeabilized cells were kept on ice until use and all mitochondria were assayed within 2 hrs of isolation.

### Mitochondrial respiration and membrane potential analysis

Permeabilized brain mitochondrial respiration was measured with an Oroboros Oxygraph 2-k high-resolution respirometry system (Oroboros Instruments, Innsbruck, Austria), as described previously [15]. Two identical respiration chambers held at either 28 or 37°C were used in parallel. Permeabilized brain cells were added to each chamber containing 2 ml of respiration solution (in mM: 0.5 EGTA, 1.4 MgCl_2_, 20 taurine, 10 KH_2_PO_4_, 20 HEPES, 1% BSA, 60 K-lactobionate, 110 sucrose; pH 7.1, adjusted with 5 N KOH). Respiratory flux through the ETS was measured in permeabilized brain cells using a substrate-uncoupler-inhibitor-titration (SUIT) protocol described previously [15, 16]. Briefly, pyruvate (5 mM), malate (2 mM), and glutamate (10 mM) were provided as carbon substrates and to spark the citric acid cycle. State III respiration flux through complex I was then achieved by adding ADP (1.25 mM). Following ADP phosphorylation, rotenone (2 μM) and succinate (10 mM) were added to inhibit complex I and assess respiratory flux through complex II. Antimycin A (2.5 μM) was then added to inhibit complex II and assess non-mitochondrial respiration. Next, carbonyl cyanide p-trifluoro-methoxyphenylhydrazone (FCCP, 1 μM), was added to fully uncouple the mitochondria and assess total ETS respiration. Finally, the electron donor tetramethyl-*p-*phenylene-diamine (TMPD, 0.5 mM) and ascorbate (2 mM) were added to the chamber to assess respiratory flux through complex IV. State II respiration was used as a proxy for leak respiration because native ATPases prevent the establishment of steady state IV respiration in the saponin-permeabilized cell preparation. Respiration values were obtained from steady-state conditions following each chemical addition. Protein content was analyzed using the Bradford technique [26].

### Proton leak measurements

Proton flux kinetics across the inner mitochondrial membrane were assessed by simultaneous measurement of oxygen consumption and H^+^-motive force, in the presence of succinate and oligomycin, using tetraphenylphosphonium (TPP^+^), as described previously [15] and using an O2k TPP^+^ ion selective electrode (Oroboros). Note that this methodology excludes any contribution of ΔpH and therefore resulting measurements of the H^+^-motive force are likely slight underestimates of the true value. Mitochondrial matrix volume was not measured because changes in this parameter have minimal impact on TPP-mediated measurements of ΔΨ_m_ [27]. We employed a binding factor b for mitochondria of 0.16 [15, 28]. The kinetics of H^+^ flux were determined by inhibiting the substrate oxidation component by stepwise addition of 0.5 or 1.0 μl aliquots of malonate (2.0 M stock) and measuring the effect on ΔΨ_m_. After the final malonate addition, FCCP (1 μM) was added to uncouple mitochondria and determine the degree of electrode drift. Proton flux curves were fit using a two-parameter exponential growth curve. A limit of the permeabilized brain preparation is that it usually presents high non-specific binding, which may vary with temperature, and is therefore not well suited to determine accurate values of ΔΨ_m_. Therefore, in the present study we focused on the comparison of mitochondrial conductance and kinetics at the two different temperatures and not the absolute values of membrane potential.

### Enzyme activities

Citrate synthase (CS) and ETS complex maximal activities (V_max_) were assessed using spectrophotometric biochemical assays from whole brain, as described previously [15], except that V_max_ was determined at either 28 or 37°C. Then, Q_10_ ratios were calculated from complex V_max_ data using the formula Q_10_ = (R2/R1)^10/T2-T1^, where R was the V_max_ of a given enzyme at a given temperature, and T represented the two experimental temperatures at which V_max_ was assayed.

### Statistics

Statistical analysis was performed using commercial software (SPSS 15.0, SPSS Inc., Chicago, IL). For all experiments, individual *n* values correspond to a single animal. Values are presented as mean ± SEM. All data were normally distributed with equal variance (*P* > 0.05). For respirometry data, significance was evaluated using a repeated-measures 2-way ANOVA to test for significant interactions between the two independent variables: (*i*) the substrate or inhibitor injected (*e*.*g*. ADP vs. succinate), and (*ii*) the experimental temperature. Bonferonni post hoc multiple comparisons tests were run on each of the dependent variables to compare the single point means of interest. For total FCCP-uncoupled respiration, non-mitochondrial oxygen consumption, and V_max_ data, significance was assessed using a student’s t-test. *P* < 0.05 was considered to achieve statistical significance unless otherwise indicated.

## Results

### ETS flux is lower in mouse brain at a hypothermic temperature relative to physiological temperature

**Fig 1:**
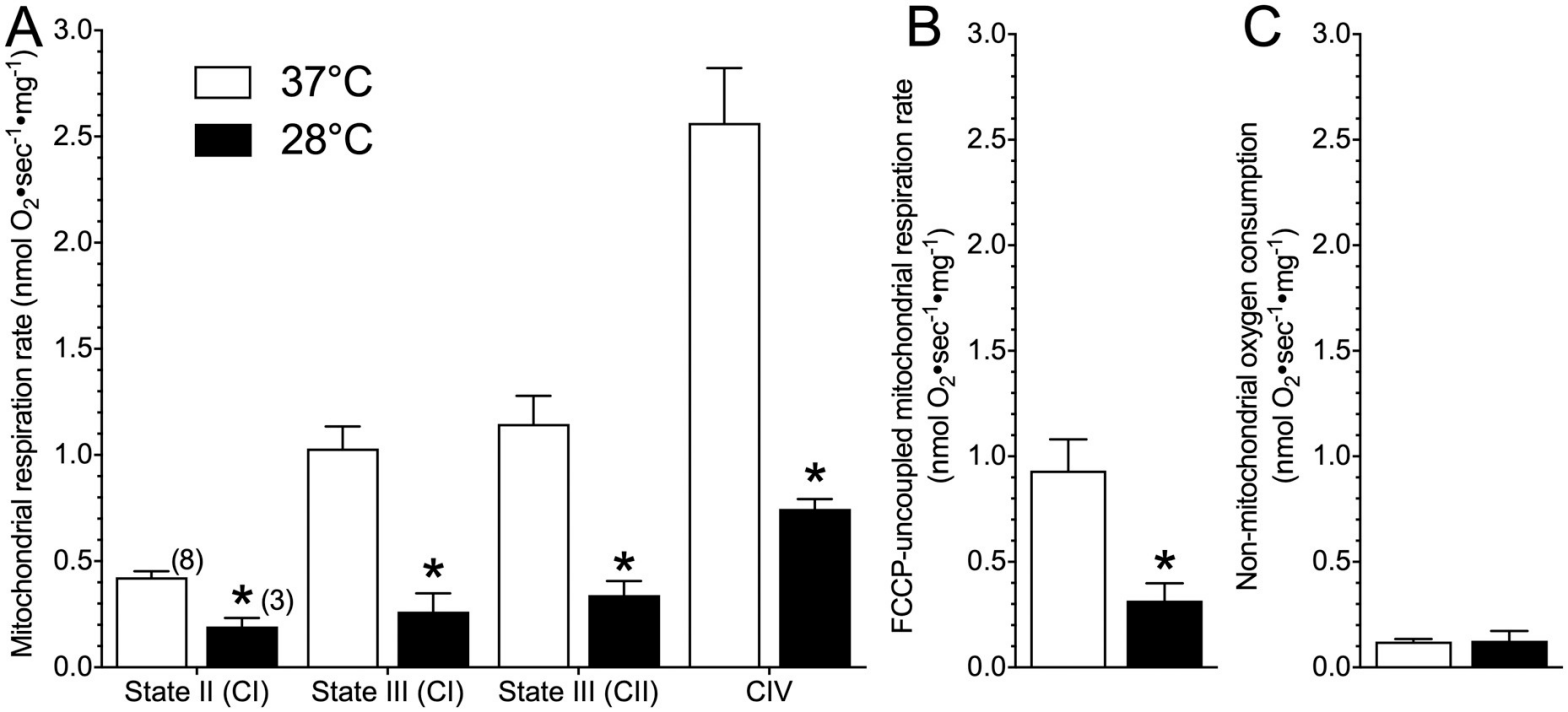
Mouse brain mitochondria have lower respiratory flux at a hypothermic temperature relative to physiological temperature. **(A)** State II (through complex I; pyruvate, malate and glutamate-fueled), state III (through complex I, ADP-fueled), state III (through complex II, succinate-fueled in the presence of rotenone), and complex IV respiratory rates from permeabilized brain mitochondria treated at physiological temperature (37°C; white bars) or a hypothermic temperature (28°C; black bars) normalized per mg protein in brain. **(B)** Comparison of complex I and II fueled FCCP-uncoupled respiration rates between experimental temperatures. **(C)** Comparison of non-mitochondrial oxygen consumption between experimental temperatures. Data are mean ± SEM. Numbers in parenthesis indicate *n*. Asterisks (*) indicate significant differences between physiological and hypothermic temperatures (p < 0.05; see Results section for statistical tests).

We examined mitochondrial respiratory flux in permeabilized mouse brain using a SUIT protocol, at the physiological temperature of mice (37°C) and at a clinically-relevant hypothermic temperature (28°C). In permeabilized brain treated at the hypothermic temperature, respiratory flux through the ETS was decreased relative to permeabilized brain treated at the physiological temperature (Fig 1). Specifically, a 2-way repeated measures ANOVA revealed a significant treatment effect between experimental temperatures on ETS respiratory flux (*F*_3,30_ = 15.0, *p <* 0.0001). Further analysis with Bonferroni posthoc tests revealed specific changes in state II respiration (with 5.0 mM pyruvate, 2.0 mM malate and 10.0 mM glutamate), and the activities of complexes I, II and IV, which were each ~ 60–65% lower in permeabilized brain at 28°C relative to at 37°C (Fig 1A; Bonferroni *p* = 0.0467 for state II, 0.0234 for CI, > 0.0161 for CII, and < 0.0001 for CIV). As a result of this consistent downregulation of the ETS, total ETS capacity (as indicated by complex I and II fueled, FCCP-uncoupled respiration rates) was reduced by ~ 67% in 28°C (Fig 1B; t(8) = 2.592, *p* = 0.032). Non-mitochondrial oxygen consumption was determined following injection of antimycin A and was not significantly different between experimental temperatures (Fig 1C; t(8) = 0.053, p = 0.9589). Based on these respiration values we also calculated Q_10_ ratios for each respiratory state. The Q_10_ ratios for each respiratory state were relatively similar and ranged from 2.61 ± 0.09 (State II respiration) to 3.92 ± 0.31 (State III respiration) (Table 1).

**Table 1:** Q_10_ values for permeabilized brain respiration rates of mouse brain. Data were calculated from respiration rates of permeabilized brain tissue at both 28 and 37°C (Fig 1). Data are mean ± SEM for *n* = 3–8 each.

Respiration State/Enzyme	Temperature Coefficient (Q_10_)
State II	2.61 ± 0.09
State III	3.92 ± 0.31
Complex V	3.09 ± 0.41
Maximum ETS capacity (FCCP-uncoupled)	3.31 ± 0.48

### ETS complex enzyme activity is generally decreased at a hypothermic temperature

**Fig 2:**
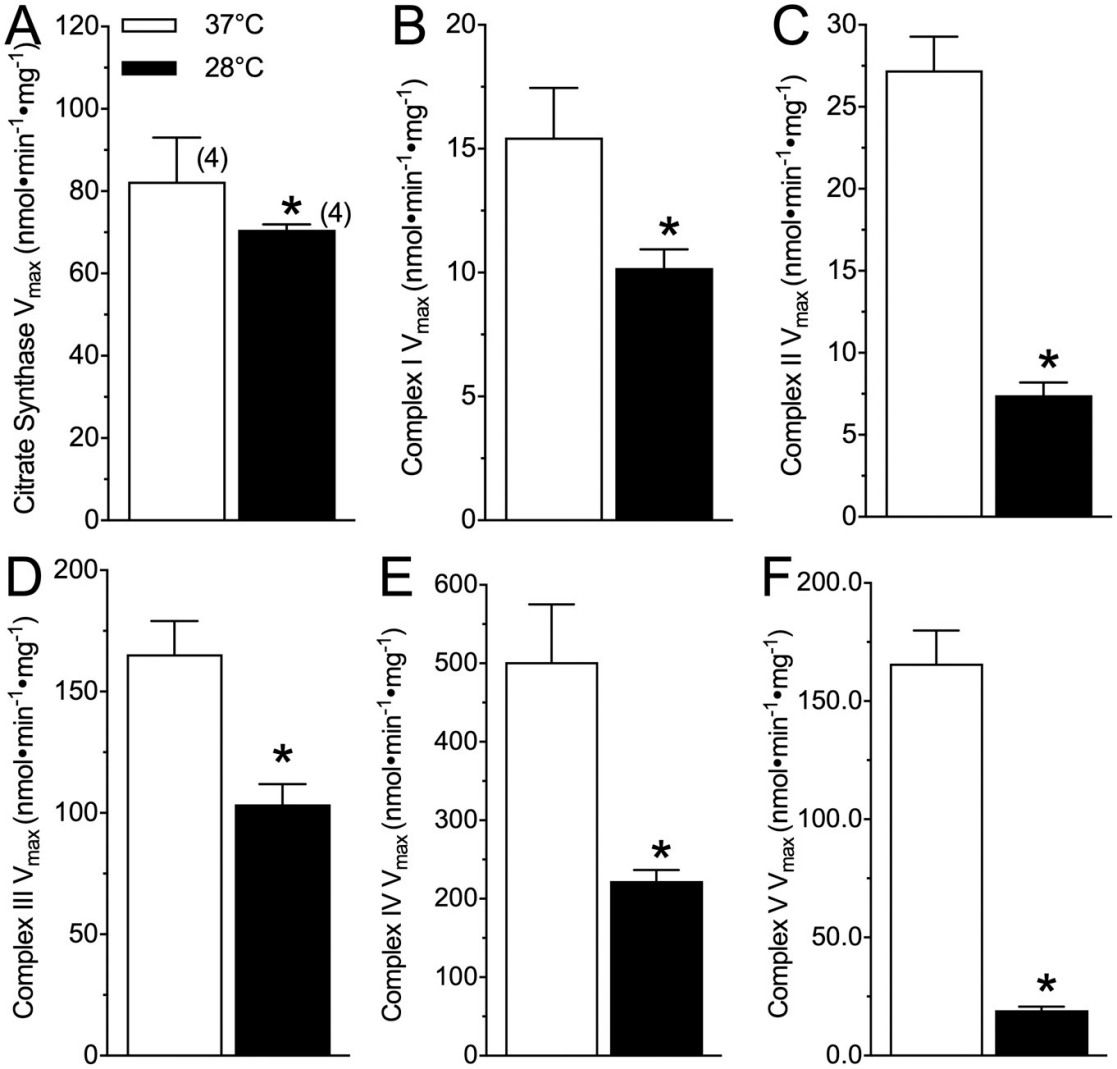
Complex enzyme maximum activity is not consistently altered by hypothermic experimental temperatures. **(A-F)** Citrate synthase (A), and complexes I (B), II (C), III (D), IV (E), and V (F) V_max_ from homogenized whole mouse brain assayed at physiological temperature (37°C; white bars) or a hypothermic temperature (28°C; black bars). Data are mean ± SEM. Numbers in parenthesis indicate the number of samples assayed from individual animals. Asterisks (*) indicate significant differences between physiological and hypothermic temperatures (p < 0.05; see Results section for statistical tests).

We next sought to more closely examine the effect of temperature upon mitochondrial function in mouse brain by examining the V_max_ of CS and complexes I-V of the ETS at our two experimental temperatures (Fig 2). Citrate synthase V_max_ was slightly decreased by ~ 14% in the hypothermic experiments, relative to physiological controls (Fig 2A; t(3) = 4.535, p = 0.021). Similarly, the V_max_ of ETS complexes I (t(3) = 2.473, p = 0.0483), II (t(3) = 7.913, p = 0.0005), III (t(3) = 3.848, p = 0.0183), IV (t(3) = 3.717, p = 0.0099), and V (t(3) = 12.36, p = 0.0001) were all decreased in the hypothermic experiment by 35, 73, 38, 55, and 88%, respectively (Fig 2B–2F). Based on these V_max_ values we also calculated Q_10_ ratios for CS and the ETS complexes (Table 2). This analysis revealed that complex II and V were particularly temperature-sensitive.

**Table 2:** Q_10_ values for citrate synthase (CS) and ETS complexes I-V of whole mouse brain. Data were calculated from enzyme V_max_ assayed at both 28 and 37°C (Fig 2) and normalized to CS activity assayed at 37°C. Data are mean ± SEM for *n* = 4 each.

Enzyme	Temperature Coefficient (Q_10_)
Citrate Synthase	1.23 ± 0.03
Complex I	1.58 ± 0.24
Complex II	4.23 ± 0.42
Complex III	1.70 ± 0.18
Complex IV	2.44 ± 0.34
Complex V	11.30 ± 0.23

### The H^+^ gradient of mouse permeabilized brain is more tightly coupled with mitochondrial respiration at a hypothermic temperature than at physiological temperature

**Fig 3:**
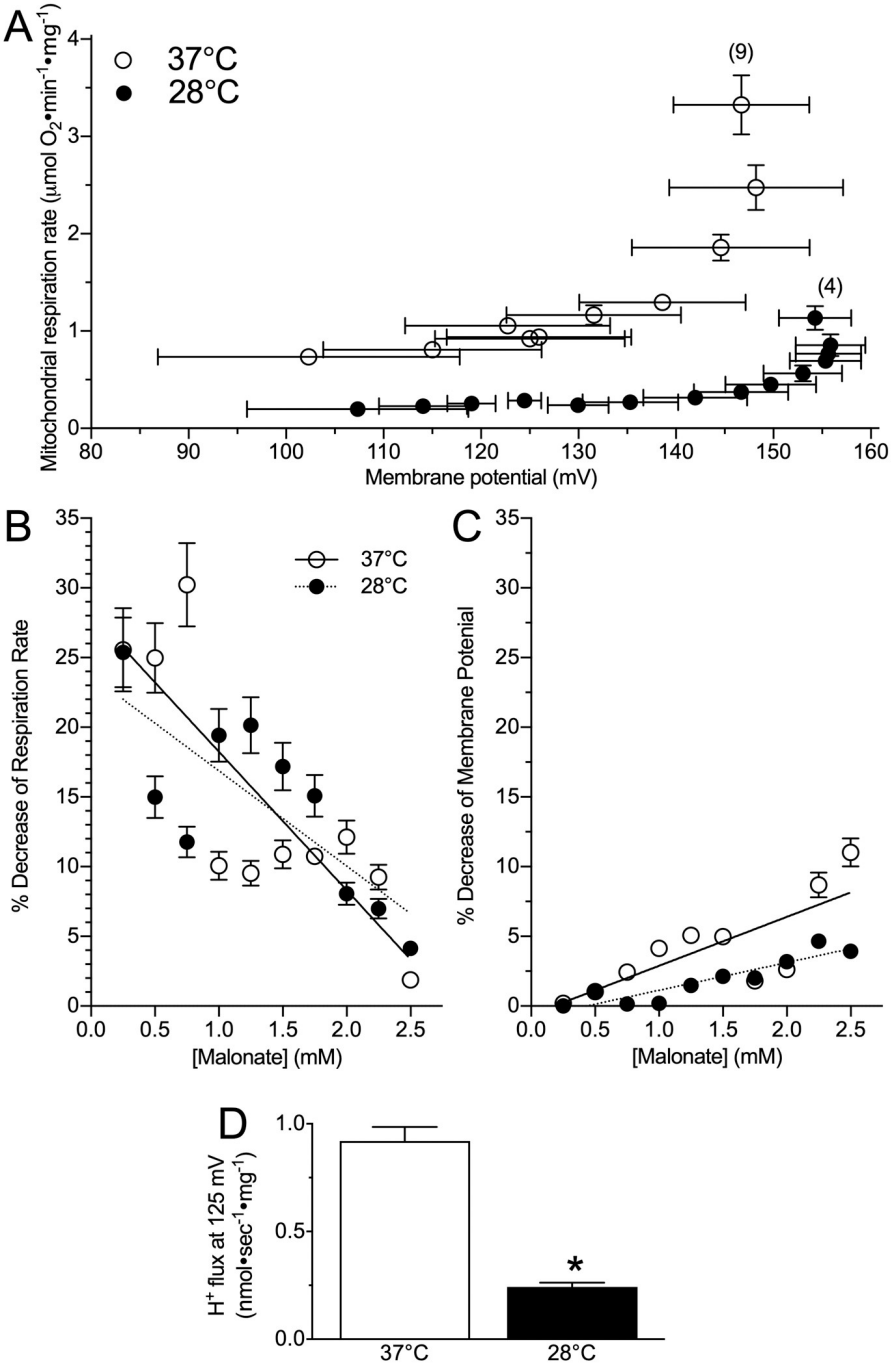
The mitochondrial H^+^ gradient of hypothermic brain mitochondria is reduced relative to physiological temperature. **(A)** Hypothermic (black circles) brain H^+^ flux and oxygen consumption are equally coupled but reduced in magnitude relative to at physiological temperature (open circles). **(B)** Percent decrease of mitochondrial respiration, and **(C)** mitochondrial membrane potential with step-wise addition of malonate. **(D)** Comparison of H^+^ flux rates at a common mitochondrial membrane potential of 125 mV. The temperature co-efficient (Q_10_) for H^+^ flux at 125 mV is 4.4. Data are mean ± SEM. Numbers in parenthesis indicate *n*. Asterisks (*) indicate significant differences between mice and NMR mitochondria (p < 0.05; see Results section for statistical tests).

We compared kinetics of the mitochondrial H^+^ gradient between experimental temperatures. A 2-way repeated measures ANOVA revealed that permeabilized brain had lower rates of oxygen consumption and a more depolarized ΔΨ_m_ at 28°C than at 37°C (Fig 3A; *F*_1,10_ = 357.0, *p <* 0.0001 for oxygen consumption and *F*_1,10_ = 24.1, *p <* 0.0001 for ΔΨ_m_). Specifically, at physiological temperatures, the maximum state II respiration rate of mouse permeabilized brain was nearly 3-fold greater than in the hypothermic temperature (Fig 3A, prior to the first malonate addition; t(3) = 12.53, p < 0.0001). When respiration was subsequently inhibited by serial additions of malonate, marked reductions in permeabilized brain respiration rates were observed at both 28 and 37°C (Fig 3A; *F*_1,10_ = 54.5, *p <* 0.0001 for effects due to malonate). However, the rates at which oxygen consumption decreased and Ψ_m_ discharged with malonate additions was not affected by assay temperature (Fig 3B and 3C; *F*_1,3_ = 1.03, *p =* 0.3254 for oxygen consumption, *F*_1,3_ = 2.096, *p =* 0.1670 for Ψ_m_ discharge). As a result of these matched reductions in respiration rates across experimental temperatures with malonate additions, respiration rates in partially inhibited mitochondria at physiological temperature remained elevated several-fold higher than in equally inhibited samples from hypothermic experiments. For example, when H^+^ flux was considered at a common Ψ_m_ of 125 mV, H^+^ flux at 28°C was ~ ¼ that at 37°C (Fig 3D; t(3) = 10.04, p > 0.0001). This retention of lower rates of respiration relative to Ψ_m_ in hypothermic conditions suggests that mouse permeabilized brain respiration was more tightly coupled with Ψ_m_ at the hypothermic temperature than at the physiological temperature.

## Discussion

We compare the functional characteristics of hypoxia-intolerant murine brain mitochondria between a physiological temperature (37°C) and a clinically-relevant therapeutic hypothermia temperature (28°C). We report that at 28°C, murine permeabilized brain has lower ETS respiration rates (Fig 1) and maximum complex activities (Fig 2) than under physiological conditions, and that less oxygen consumption is required to maintain a specific charge across the mitochondrial membrane in the therapeutic hypothermia temperature (Fig 3). This finding indicates that mitochondrial respiration is more tightly coupled to the H^+^ gradient in the therapeutic hypothermia temperature and that mitochondria are therefore more efficient in hypothermia. Furthermore, we report that in permeabilized murine brain, the temperature coefficient of mitochondrial respiration falls within a range of ~ 2.5–4.0 (Fig 2, Table 1). To our knowledge, our study is the first to directly examine the effect of temperature on mitochondrial function in mice but a handful of studies have examined Q_10_ effects on isolated mitochondrial function in squirrel brown adipose tissue, muscle, and liver and our *in vitro* respiration data agree reasonably well with these previous mammalian studies. For example, in squirrel brown adipose tissue (measured between 10–37°C), the Q_10_ of state II respiration is 2.4 [29], which agrees well with our finding in mouse brain (2.6). Similarly, we report a Q_10_ value of 3.9 for murine brain State III respiration, which falls reasonably between the reported Q_10_ values for State III-respiring muscle (~ 2.8) and liver (~ 6.5) mitochondria, isolated from summer active squirrels and measured between 25–37°C [30].

Overall, we report an *in vitro* temperature coefficient for murine brain ETS respiratory flux (in FCCP-uncoupled permeabilized brain) of 3.3, indicating that this tissue is moderately temperature-sensitive. This sensitivity may be expected to confer significant metabolic rate reduction-related energy savings at the colder temperature, which likely contributes to enhanced neuroprotection during hypoxia or ischemia in therapeutic hypothermia-treated subjects. Indeed, reducing metabolic demand when oxygen is limiting, particularly in brain tissue, is a common strategy employed by hypoxia-tolerant and hypoxia-adapted species [31], including in human populations that have lived at altitude for 1000’s of years [32]. Moreover, the Q_10_ of H^+^ leak at a common resting membrane potential (125 mV) is 4.4, indicating that murine brain H^+^ leak decreases faster than does respiration rate with decreasing temperatures. Greater reductions in H^+^ leak indicate enhanced mitochondrial efficiency and are expected to confer further metabolic savings in hypoxia or ischemia, thereby likely contributing to the neuroprotective effect of therapeutic hypothermia treatment.

Relative to *in vitro* respiration measurements from permeabilized brain, *ex vivo* whole brain assays of enzyme V_max_’s reveal more varying thermal sensitivity in mitochondrial function (Fig 2; Table 2). Specifically, CS and ETS complexes I, III, and IV are relatively temperature-insensitive (Q_10_’s 1.2–2.4), whereas complex II (4.2), and particularly, complex V (11.3) are highly sensitive to temperature changes. The greater temperature sensitivity (*i*.*e*., higher Q_10_ values) between our whole brain and permeabilized brain measures of complex V activity may be due to reduced manipulation and degradation of the whole brain sample relative to permeabilized brain samples, and thus the V_max_ data is likely more representative of *in vivo* flux potential for each complex.

This profile of temperature sensitivity would favour a switch towards CI-mediated ATP-production, which is considerably more oxygen-efficient than CII-mediated ATP production [33]. Such a switch may contribute to increased mitochondrial efficiency in the hypothermic temperature because the P/O ratio associated with the oxidation of NADH-linked substrates by complex I is ~ 2.5, whereas the ratio for oxidation of succinate by complex II is ~ 1.5 [33]. Similarly, the H^+^/O ratio is more efficient for complex I respiration (~ 10) than for complex II respiration (~ 6) [33]. Maximizing energy yield from limited oxygen availability in hypoxia is of obvious benefit and these temperature coefficients suggest that therapeutic hypothermia may confer energetic-efficiency benefits in hypoxia by preferentially down-regulating CII-mediated respiration in favour of CI-mediated respiration. In addition, and relative to the overall inhibition of the ETS in the therapeutic hypothermia temperature, the larger magnitude reduction in the activity of complex II may minimize deleterious ROS generation during reperfusion that has been linked to succinate accumulation during ischemia [34], whereas the significant reduction in complex V activity may help to minimize ATP consumption through reverse-flow of the ATPase [35], and thus contribute to the retention of energy stores during low oxygen stress.

### Study limitation

An important caveat of our study concerns the experimental temperatures employed. Specifically, a recent study has demonstrated that mitochondria *in situ* are ~ 10°C warmer than physiological temperatures and that V_max_ activity is maximized at ~ 50°C [36]. However, in isolated mitochondrial preparations, samples do not tolerate such high temperatures well and therefore we conducted our control experiments at the typical murine body temperature of ~ 37°C, which is also consistent with the majority of murine mitochondrial studies.

### Conclusions

Mitochondria are the nexus of oxygen consumption and metabolism in the cell, and also coordinate deleterious and neuroprotective responses to low oxygen stress [18]. Our findings support a role for remodelling of mitochondrial energetics during therapeutic hypothermia that may contribute to reduced tissue-specific metabolic rate and energy savings that would in turn limit excitotoxicity, ROS generation, and cell death pathway initiation. Hypoxia-tolerant species typically have low resting body temperatures and thus, therapeutic hypothermia may provide neuroprotective benefits to hypoxia-intolerant species that are endogenously available to animals with lower metabolic rates and body temperatures by enhancing the efficiency of mitochondrial respiration.
